# Phytohormonal Regulation of Abiotic Stress Tolerance, Leaf Senescence and Yield Response in Field Crops: A Comprehensive Review

**DOI:** 10.3390/biotech14010014

**Published:** 2025-02-27

**Authors:** Anna Panozzo, Pranay Kumar Bolla, Giuseppe Barion, Alessandro Botton, Teofilo Vamerali

**Affiliations:** Department of Agronomy, Food, Natural Resources, Animals and the Environment, University of Padua, Viale dell’Università 16, 35020 Legnaro, PD, Italy; pranaykumar.bolla@unipd.it (P.K.B.); giuseppe.barion@unipd.it (G.B.); alessandro.botton@unipd.it (A.B.); teofilo.vamerali@unipd.it (T.V.)

**Keywords:** Cytokinins, gibberellic acid, abscisic acid, drought, plant senescence

## Abstract

Field crops are expected to be increasingly threatened by climate change, which will negatively impact plant development, growth and yield. Phytohormones play a crucial role in regulating specific signalling pathways to induce rapid adaptive responses to environmental stresses. Exogenous phytohormone application alters hormonal balance, thereby enhancing plant adaptation to adverse conditions. While several studies have advanced our understanding of the use of phytohormones in field crops, yield responses and species-specific application strategies remain inconsistent and rarely assessed under field conditions. The application of cytokinins (CKs), abscisic acid (ABA), and gibberellic acid (GA) has been shown to maintain prolonged photosynthetic activity, stabilize plasma membrane, and reduce lipid peroxidation and ion accumulation under salinity stress in wheat. Additionally, inhibitors of ethylene synthesis and receptors can mitigate stress symptoms under drought and heat stress, which typically accelerates senescence and shortens the grain-filling period in cereal crops. In this way, exogenous application of CKs, GA, and ethylene inhibitors can delay senescence by sustaining leaf photosynthetic activity and postponing nutrient remobilization. However, these benefits may not consistently translate into improvements in grain yield and quality. This review explores the molecular mechanisms of phytohormones in abiotic stress tolerance, delineates their specific functions and evaluates experimental findings from field applications. It also summarizes the potential of phytohormone applications in field crops, emphasizing the need for species-specific investigations on application timing and dosages under open-field conditions to optimize their agronomic potential.

## 1. Introduction

Global food security is a critical concern in light of the rapidly growing global population [1]. As climate change increases, crop production faces significant challenges due to abiotic stresses—such as drought and salinity, and even flooding—that are expected to become more severe and widespread in the near future. Within this framework, it is imperative to enhance field crop mechanisms to adapt to various suboptimal environmental conditions, ensuring the development of stress-tolerance responses and maintaining high yield and quality standards.

Crop yield is the result of efficient photosynthesis after anthesis, nutrient accumulation during the vegetative phase, and the efficient translocation from leaves to developing grains during grain filling. This process is heavily influenced by environmental factors such as temperature, water availability and nutrient content to which plants are exposed [2]. Consequently, as rainfall patterns and temperature dynamics are increasingly impacted by climate change, there is an urgent need to develop strategies that enable plants to overcome environmental constraints [3]. Abiotic stresses are considered the predominant cause of crop losses worldwide, reducing the yield of the major field crops by more than 50% [4]. Environmental conditions that impact plant growth and reproductive capacity include drought, flooding, temperature extremes, nutrient deficiency, metal toxicity and pathogen infection [5]. Plants have evolved various mechanisms to perceive incoming stresses and rapidly regulate their physiology and metabolism to cope with them [6]. This regulation is mediated by plant phytohormones, a group of naturally occurring organic substances that exert significant influence on physiological processes even at very low concentrations [7].

Phytohormones are vital in integrating environmental signals into plant development. They are essential for detecting stressors, incorporating these signals into endogenous signaling pathways, and promoting adaptation strategies. Additionally, through both independent and interactive (crosstalk) pathways, they coordinate the senescence process. The timing and progression of senescence determine the effective remobilization of assimilates from leaves to grains, thereby influencing the yield and quality of many crops. The application of exogenous phytohormones has the potential to alter the endogenous hormonal balance in cultivated plants, enhancing their adaptation to stress conditions, especially during the grain-filling period. Despite being well-known and widely used in fruit tree management, phytohormones have received relatively little attention in field crop agricultural practices. Historically, adaptation to various environmental stresses in field crops has primarily focused on conventional breeding techniques for tolerance traits and molecular genetic mutations [8,9].

The potential of phytohormones to enhance resilience and yield in field crops is an area of great interest. Specific phytohormones can exogenously modulate the tolerance response in crops and mitigate the after-effects of abiotic stresses. It is expected that elevated levels of certain hormones in plant tissues will promote environmental stress defense and regulate the grain-filling rate, thereby enhancing crop resilience. However, our understanding of the utilization of these molecules in field crops remains limited and not comprehensively summarized. The practical application of phytohormones faces several challenges, such as determining the optimal concentration and timing of application in relation to crop response, environmental conditions, and the crosstalk between multiple phytohormones. Nonetheless, the beneficial potential of phytohormones in agriculture is significant, warranting further research in these areas.

Given this context, the purpose of this review is threefold: (i) to review the molecular basis of phytohormone regulation in field crops, (ii) to provide a current overview of the role of phytohormones in regulating signaling pathways involved in adaptation responses to abiotic stresses and senescence dynamics, and (iii) to summarize the main experimental results to date on the use of exogenous phytohormones to enhance abiotic stresses tolerance and regulate senescence in relevant field crops.

## 2. The Molecular Basis of Phytohormone Regulation Activity

Apart from auxins, the main phytohormones involved in regulating plant growth and development are cytokinins, abscisic acid, ethylene, gibberellins, jasmonates, and salicylic acid. These phytohormones play a pivotal role in translating stress-related signals into alterations of gene expression, thereby inducing appropriate adaptation responses to suboptimal environmental conditions [5]. This hormonal regulation of plant growth and development is achieved not only by modulating hormonal levels in response to changes in the gene transcription but also by directly regulating gene expression [7], often in interplay with other hormonal pathways. One such pathway includes grain protein content (*Gpc*) genes and DELLA proteins, which are repressors in the gibberellin signal transduction pathway and are also known to be responsive to cytokinin levels in plant growth and stress [10].

Notably, in cereals like wheat and barley, *Gpc* genes represent one of the most thoroughly investigated case studies for senescence regulation [10]. In the context of nutrient remobilization to grains, *Gpc-B1* emerged as the pioneer gene responsible for variation in grain protein content (GPC) identified through a map-based cloning approach in wheat. With the advancements of genetic methodologies, particularly in model organisms like *Arabidopsis thaliana*, our knowledge of phytohormone biosynthesis, metabolism, transport, perception, signaling and response has exponentially grown over the past few years.

### 2.1. Cytokinins

Cytokinins (CKs) are adenine-derived compounds that regulate various aspects of plant growth and development and are crucial for stress adaptation [11]. These compounds are produced in root tips and developing seeds [12,13], and they are translocated to shoots through the xylem, a process facilitated by the transpiration stream. This mechanism is proposed to integrate shoot and root physiology as well as to regulate developmental processes and senescence [5]. Cytokinin biosynthesis is catalyzed by the isopentenyl transferase (IPT) enzyme, encoded by a multigene family [14,15]. The degradation of CKs is mediated by CK dehydrogenases (CKXs), enzymes that remove unsaturated isoprenyl side chains of CKs [11]. Seven *CKX* genes (*CKX1 to CKX7*) have been identified in *Arabidopsis*, each encoding different isoenzymes with distinct expression patterns. Overexpression of *CKX* genes causes a decrease in CK levels. The cytokinin signaling pathway involves two-component regulators acting as receptors [16]. CK receptors are hybrid histidine kinases (HKs), and the *Arabidopsis* genome encodes six HKs: AHK1, AHK2, AHK3, AHK4, AHK5/CKI2 and CKI1 [17]. Each receptor plays a distinct role in regulating plant growth and development and exhibits varying localization and sensitivity to CKs. CKs were shown to inhibit leaf senescence when their biosynthesis genes are driven by the SAG12 promoter, a common marker for developmental leaf senescence [18]. Moreover, cytokinins have been reported to affect stress tolerance in a peculiar way. For instance, a constitutive downregulation of CK levels, either through the overexpression of *CKX* genes or the inactivation of *IPT* genes, results in a drought- and salt-tolerant phenotype, most likely because of the reduced growth rate associated with lower energy consumption [11]. On the other hand, higher CK levels in transgenic tobacco overexpressing CK biosynthesis genes fused with a stress-responsive promoter resulted in drought tolerance. In this case, the tolerant phenotype was due to the prolonged maintenance of the photosynthetic machinery and increased root growth relative to shoot growth [19]. These apparently contradictory results support multiple roles for CKs in stress response, both when their levels are lower and higher than normal. Concerning the specific involvement of CK receptors in stress responses, while the AHK1 receptor has been recognized as a positive regulator of drought and salt stress responses and abscisic acid (ABA) signaling, its homologous receptors AHK2, AHK3 and CRE1 act as negative regulators in ABA signaling and negatively control the stress responses [20]. These data further support a multifaceted role for cytokinins in stress response.

### 2.2. Abscisic Acid

Abscisic acid (ABA) is a sesquiterpenoid phytohormone that plays a key role in regulating seed dormancy, stomatal closure, the shoot-to-root growth ratio, leaf senescence and responses to biotic and abiotic stresses [18]. ABA is an important isoprenoid hormone produced by the plastidial 2-C-methyl-D-erythritol-4-phosphate (MEP) pathway [21]. The receptors involved in its signaling pathway include G-protein-coupled receptors and chelatase [22]. A variety of receptor candidates have been proposed in the literature. Shen et al. [23] highlighted the role of magnesium protoporphyrin-IX chelatase H subunits (CHLH) in ABA activity [24]; however, studies on mutants with reduced CHLH levels did not consistently display ABA-related phenotypes [25]. GCR2, a G-protein-coupled receptor, has also been suggested as a candidate ABA receptor [26]. More recently, another pair of G-protein-coupled receptors, GTG1 and GTG2, were implicated in ABA responses [27]. Key players in ABA signal transduction include the type 2C protein phosphatases (PP2Cs) ABI1 and ABI2, which negatively regulate ABA responses. Ma et al. [28] identified interactors of ABI1 and ABI2, which they termed regulatory components of ABA receptor (RCARs). By examining RCAR proteins from different clades within the family, Ma et al. [28] found that 14 members might serve as ABA receptor components. This 14-member RCAR gene family was independently identified by Park et al. [29], who analyzed genes involved in ABA signaling and named them *Pyrabactin Resistance* (*PYR1*) and *PYR1-Like* (*PYL1*). The *PYR*/RCAR system is now recognized as the most important ABA receptor system. Different RCARs have been shown to bind ABA, mediate ABA-dependent inactivation of ABI1 or ABI2 in vitro, and regulate ABA responses [28].

Several recent studies have reported that ABA is involved in the negative regulation of plant defense responses. Xing et al. [30] showed that ABA induces the expression of catalase (CAT1), which scavenges H_2_O_2_, while also activating H_2_O_2_ production, suggesting H_2_O_2_ involvement in ABA-induced CAT1 expression. Moreover, ABA’s involvement in the leaf senescence process is linked to changes in ABA signaling that induce transcription of SAG113, a gene that is specifically expressed during senescence; overexpression of SAG113 resulted in more rapid water loss and accelerated leaf senescence [19,31].

### 2.3. Ethylene

Ethylene is a gaseous phytohormone that affects various processes during plant development, including cell division, cell elongation, cell size, climacteric fruit ripening, and the senescence process [19,32]. It plays an important role in acclimation to both biotic and abiotic stress by modulating tissue sensitivity and plant responses [33,34]. Ethylene signaling primarily depends on the repression of the function of the five ethylene receptor genes—*Ethylene Response1* (*ETR1*), *Ethylene Response Sensor1* (*ERS1*), *ETR2*, *Ethylene Insensitive4* (*EIN4*) and *ERS2*—which otherwise repress ethylene responses via the negative regulator *Constitutive Response1* (*CTR1*) [35]. Subsequently, ethylene responses are positively regulated by *Ethylene Insensitive2* (*EIN2*) and *3* (*EIN3*), as well as by downstream primary and secondary ethylene-responsive genes such as *Ethylene Response Factor1* (*ERF1*) genes [36]. EIN3 levels are regulated by a two-component receptor system. According to Guo and Ecker [37], at least two F-box proteins, EBF1 and EBF2, regulate EIN3 levels—the first repressing EIN3 levels when ethylene is low, while the second may prevent excessive accumulation of EIN3. Recently, Qiao et al. [38] demonstrated that ETP1 and ETP2, another pair of F-box proteins, promote the degradation of ethylene signaling protein EIN2 in the absence of ethylene. The role of ethylene in controlling the timing of leaf senescence depends on the transcriptional regulator EIN2 [39], which regulates the transcript abundance of ANAC092, an ethylene-inducible positive regulator of senescence. The common target for reduced ethylene production or perception is 1-aminocyclopropane-1-carboxylic acid synthase (ACC synthase), a key enzyme encoded by a multigene family that catalyzes the rate-limiting step of ethylene biosynthesis [40].

Plants can also benefit from some endophytic bacteria that degrade ACC—the precursor of ethylene—through ACC deaminase activity. This enzymatic process produces α-ketobutyrate and ammonia, thereby lowering ACC levels and preventing excessive ethylene under various stress conditions, such as salt stress [41,42].

### 2.4. Gibberellic Acid

Gibberellins (GAs) are pentacyclic diterpenes known for their role in cell elongation, seed germination, senescence, and tolerance against various environmental stresses [43]. At least five biologically active gibberellins—namely gibberellins A1, A3, A4, A5 and A7 —play different roles in physiological contexts and plant species [44]. The molecular characterization of various GA response mutants led to the discovery of the GID1 and DELLA proteins, which are key components of the molecular GA-GID1-DELLA signaling mechanism that enables plants to respond to GA [45]. GID1 is predominantly localized in the nucleus and acts as a soluble GA receptor, with high affinity for bioactive GAs [46]. GID1 is therefore the main receptor of GA, interacting with DELLA proteins [16], which are hormone-sensitive lipase-like proteins that negatively regulate GA responses; thus, GAs promote growth by stimulating DELLA protein degradation [22]. De Lucas et al. [47] demonstrated that DELLA proteins directly interact with the DNA-binding domains of transcription factors PIF3 and PIF4, sequestering them in inactive complexes. Their study also described how GA accumulation destabilizes DELLA proteins, thereby freeing PIF3 and PIF4 to activate the transcription of their target genes. DELLA proteins have been shown to promote the expression of genes encoding reactive oxygen species (ROS) detoxification enzymes and to regulate ROS levels in response to biotic and abiotic stresses [48]. Additionally, recent studies found that *Arabidopsis* DELLA proteins control plant immune responses by modulating salicylic acid and jasmonate-dependent defense responses [49].

### 2.5. Salicylic Acid

Salicylic acid (SA) is a phenolic phytohormone that regulates seed germination, fruit ripening, and flowering. Additionally, it plays a crucial role in plant defense responses to a range of abiotic stresses, including salinity and cadmium toxicity. SA is recognized as the major regulator of reactive oxygen species (ROS), given its key role in establishing the hypersensitive response (HR) and systemic acquired resistance (SAR) [50]. It is involved in controlling the dynamics of morpho-physiological and molecular responses by transducing signals through a multifaceted network of SA receptors such as the non-expressor of pathogenesis related protein-1 (NPR1)—as well as reactive oxygen species, calcium ions, nitric oxide and mitogen-activated protein kinase cascades [50]. These downstream signaling molecules facilitate the movement of SA from the cytosol to the nucleus, leading to the upregulation of defense genes that activate plant defense mechanisms. NPR1 (also known as Non-Inducible Immunity-1 (NIM1)) is notably recognized as the chief redox regulator of SA-mediated defense signaling [51].

### 2.6. Jasmonic Acid

Jasmonic acid (JA) is a lipid hormone, belonging to a class of fatty acid derivatives, that affects seed germination, root growth, leaf aging and flowering time. The JA signaling pathway is also one of the main mechanisms of plant defense responses to biotic and abiotic stresses [52], which strongly rely on the JAZ protein family as negative regulator. Under normal conditions, the level of JA in plants is relatively low, and the JAZ protein binds to transcription factors such as MYC2 to induce a series of transcriptional suppressors and inhibit the expression of JA response genes. When plants are subjected to biotic or abiotic stress, their JA contents increase, which promotes the interaction between the JAZ repressor protein and SCF^COI1^ receptor complex, inducing the release of transcription factors that activate the expression of JA response genes [53].

Several studies have shown that the response mechanisms triggered via JA signaling are mainly exerted through the activation of the antioxidant system, the accumulation of amino acids and soluble sugars and the regulation of stomatal opening [54]. Under drought conditions, JAZ proteins activate related transcription factors, such as MYC2, which stimulate the upregulation of stress tolerance genes, improving the photosynthetic rate, stomatal conductance and the antioxidant system [55]. Endogenous upregulation of JA also enhanced salt tolerance mainly by maintaining the ROS balance and promoting Na^+^/K^+^ homeostasis [56,57]. Under high-temperature conditions, the accumulation of JA and its derivative jasmonoyl-L-isoleucine (JA-Ile) increased significantly, enhancing the soluble sugar content and ROS regulation [58,59].

## 3. Phytohormone Regulation of Field Crop Responses to Abiotic Stresses

Perception of stress cues and relay of signals to activate adaptive responses are the key steps in plant stress tolerance [60]. Crop growth reduction due to water deficiency is significant and may dominate all other factors of yield, making drought one of the major contributors to reduced field crop productivity worldwide [61]. Drought limits plant growth by causing photosynthetic decline, osmotic stress and interference with nutrient availability as the soil dries. Similarly, salinity—often associated with drought—hinders seed germination, seedling growth, enzyme activity, and protein synthesis [62,63]. Flooding alters nitrogen pathways, reduces nutrient availability and leads to plant growth impairments and grain yield losses, especially during early growth stages. Heat stress also impacts a multitude of plant and cellular functions; high temperatures are known to damage proteins through denaturation, increase pollen sterility and seed abortion, and accelerate premature senescence [64].

A common consequence of most abiotic stresses is an increased production of reactive oxygen species (ROS) [64], including the superoxide radical, hydrogen peroxide, and hydroxyl radical, which result from one, two or three electron transfers to dioxygen. These toxic ROS can damage DNA, proteins, lipids, and nearly every organic component of the cell. To mitigate this toxicity, the production of antioxidant detoxification enzymes is crucial. However, an imbalance between ROS production and antioxidant defense often induces osmotic stress that further damages plant cells [21,64]. Since these processes are activated and regulated by phytohormone activity, the reduction in plant growth under stress conditions can be largely attributed to an altered hormonal balance. Here, we summarize current literature on how phytohormones regulate various physiological processes responsible for adaptation responses—focusing primarily on drought and salinity, the two most relevant abiotic stressors affecting growth and yield in field crops.

Adaptation to drought stress is a complex process that involves the coordinated activity of multiple phytohormones and intricate crosstalk between their signaling pathways. ABA has been widely recognized as a major regulator of plant responses to water stress by several authors [16,62,65,66,67]. An increase in ABA levels in xylem tissues during water stress periods is thought to regulate the balance between water loss and uptake, as well as assimilation of carbon dioxide, thereby controlling the transpiration and osmotic processes within plant cells. Elevated concentrations of ABA were shown to coordinate stomatal closure and promote dry matter accumulation, particularly of proline, an amino acid that protects membrane structure and functions during water stress [40,68,69,70]. Fricke et al. [71] demonstrated that ABA contributes to the increased xylem water potential and water uptake in saline environments.

Cytokinins are recognized for their role in regulating several physiological responses to abiotic stresses. They strongly promote cell division, have a positive effect on photosynthesis, and stimulate sink strength and assimilate distribution [11]. The roots are the primary sites for cytokinin production, and CKs are often regarded as fundamental signals for root growth [5]. The timing of cytokinin biosynthesis and action is crucial for regulating root and shoot growth. CK metabolism is modulated dynamically: under short-term or mild stress, a transient elevation in CK levels may promote cell division; however, under prolonged or severe stress, CK levels typically decrease, leading to reduced growth and a shift in energy resources toward defense mechanisms [72,73,74]. Thus, CKs are generally considered negative regulators of stress signaling. According to Ha et al. [11], this endogenous regulation of CKs is linked to the observed reduction in growth rates in stress-affected plants, as it lowers energy consumption. Studies of CK-deficient plants have shown an increase in drought tolerance, attributed to improved maintenance of cell membrane integrity and stability under water stress conditions [74].

While individual phytohormones play major roles in regulating stress responses, the interactions and crosstalk between pathways are even more significant. These interactions lead to complex regulatory networks, allowing plants to fine-tune their response to various environmental stresses. Several studies have demonstrated that cytokinins regulate stress responses through intensive interactions with abscisic acid [11,19,75]. CKs may act as antagonists to ABA in various plant growth processes [76]; during drought, a reduction in CK content has been linked to hypersensitivity to ABA [74]. Thus, downregulation of CK signaling induces the expression of ABA-responsive genes, contributing to an enhanced salt and drought stress tolerance [77,78]. Drought adaptation has been associated with the enhancement of leaf antioxidant enzyme activities, resulting in higher concentrations of peroxidase (POD) and superoxide dismutase (SOD), but also with the suppression of lateral root formation and the promotion of primary root growth to access water of deeper soil layers [79]. Auxin signaling is modulated via the antagonistic interactions between CK and ABA under water deficit conditions. Shkolnik-Inbar and Bar-Zvi [80] reported high levels of both CKs and ABA in drought-stressed roots, noting that these compounds act as antagonists to auxin, which regulates the initiation of lateral roots [80]. In some cases, ABA and CKs may act synergistically to interact with auxin signaling pathways, thereby modulating root development in response to water deficit.

Ethylene biosynthesis and signaling activity are intricately linked to crop exposure to stress conditions. The release of ethylene from plant tissues is known to be triggered when the plant is affected by environmental stressors such as drought, salinity and high temperatures [81,82,83]. Although, the dynamics of ethylene release were widely investigated in arboriculture, an increasing body of literature now addresses field crops [84,85]. For instance, when wheat plants were subjected to heat stress (38 °C) at nine days post pollination, ethylene production in developing kernels and in the flag leaf increases by 6-fold and 12-fold, respectively [86]. Specifically, ethylene production began two hours after the onset of heat stress in developing kernels and nine to ten hours in the flag leaf. Early studies using excised segments from crop leaves subjected to different abiotic stresses also revealed these dynamics [87]. Under water stress, increased ethylene production results from higher levels of the precursor 1-aminocyclopropane-1-carboxylic acid (ACC) and increased activity of the ACC-oxidase enzyme (ACO), which catalyzes the conversion of ACC to ethylene [88]. Ethylene produced under stress plays a key role in tolerance responses by enhancing antioxidant accumulation, promoting stomatal closure, and reducing ROS accumulation. Moreover, ethylene is crucial in triggering defense response under hypoxic conditions by promoting an accelerated elongation of submerged stems, leaf petioles and the adventitious rooting on stems at or above the water line [82,89,90,91]. A substantial body of the literature has also described interactions between ethylene and gibberellins in activating acclimation responses in submerged rice [92,93]. Tolerance to submergence is coordinated by the submergence-inducible gene *Sub1A*, which encodes an ethylene-responsive factor-type transcription factor (ERF). *Sub1A* increases the accumulation of the GA signaling repressors Slender Rice-1 (SLR1) and SLR1 Like-1 (SLRL1) while diminishing GA-inducible gene expression under submerged conditions [92]. During this, *Sub1A* limits ethylene-promoted GA responsiveness by augmenting accumulation of the GA signaling repressors SLR1 and SLRL1 [93].

The promotion of ethylene production under stress conditions is also proposed to be regulated by other hormone signaling pathways, particularly through the crosstalk with ABA. Sharp [94] and Iqbal et al. [88] highlighted the interaction between ABA and ethylene in modulating root and shoot growth responses to water stress. This involvement of ABA in the mechanism of differential sensitivity towards shoot and root growth has been receiving attention only during recent times, mainly on ABA-deficient plants [95,96]. ABA was found to accumulate in shoot and root tissues under water-limited conditions and to correlate with growth inhibition. Moreover, when ABA was applied to well-watered plants, the effects of growth reduction were the same as those resulting from endogenous ABA accumulation [83]. However, a study on ABA-deficient maize seedlings showed an important role of endogenous ABA in maintaining primary root growth during drought and in the prevention of excess ethylene production [97]. In ABA-deficient maize seedlings under water stress, ethylene production was significantly higher than in control plants, but this effect was reversed when the root ABA content was restored with exogenous ABA, together with restoration of root elongation. Additionally, the magnitude of the ethylene increase correlated with both the degree of ABA deficiency and the inhibition of root elongation [97].

ABA is often considered as an inhibitor of shoot growth, mainly based on short-term studies at low water potential [83,98]. In maize seedlings subjected to water stress, ABA accumulation initially inhibited shoot growth, but after 70 h, increased ABA levels appeared to help maintain shoot growth [97]. While optimal ABA levels sustain growth, the endogenous ABA concentrations in shoots may be insufficient for maximal growth in later stages of water stress. Based on these findings, Sharp [94] proposed that endogenous ABA helps maintain rather than inhibit shoot growth in water-stressed plants over the long term. Nonetheless, the accumulation of ABA may not fully counteract ethylene-induced growth inhibition, contributing to the higher sensitivity of shoot growth compared to root growth.

Phytohormones also play an integral role in the regulation of plant adaptation to oxygen deficiency, as a majority of morphological and metabolic adaptations during flooding are strictly regulated by hormone pathways. Oxygen deficiency triggers ethylene production, which promotes the development of adventitious roots and lysigenous aerenchyma. ACC is synthesized in hypoxic roots and transported via xylem sap to aerated shoots, where it is converted into ethylene. Flood-induced ethylene production further leads to a decreased levels of ABA and gibberellins [99].

Regarding GAs and SA, these hormones have a minor yet significant role in stress signaling pathways. GAs accumulate rapidly when plants are exposed to abiotic stresses and this accumulation has been reported to be relevant in enhancing wheat and rice growth under saline conditions [62,88,100,101,102]. Similarly, SA levels increase during biotic stress, contributing to various tolerance mechanisms through enhanced antioxidative capacity and stimulation of RUBISCO activity [16,103,104,105,106].

## 4. Exogeneous Phytohormone Application for Adaptation to Abiotic Stress

**Figure 1:**
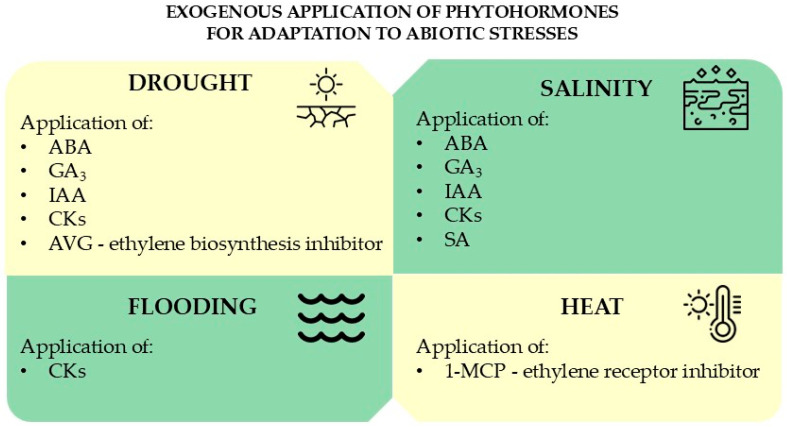
Exogenous application of phytohormones involved in cereals’ adaptation.

In the literature, studies investigating how exogenous application of phytohormones affects plant stress tolerance in field crops were mostly conducted under controlled laboratory or greenhouse conditions. In this section, we summarize current scientific findings on the exogenous application of phytohormones to mitigate the impact of drought, salinity, flooding, and heat stress in field crops. Various methods of phytohormone application are reported, depending on the phytohormone being tested, environmental stress and the crop species. These methods include (i) pre-sowing seed treatment, (ii) foliar application during vegetative growth or (iii) post-anthesis supply to act on senescence timing and abiotic stress tolerance (Figure 1, Table 1).

### 4.1. Phytohormone Application and Drought

Osmotic stress due to drought or salinity is one of the most threatening abiotic stresses in field crops worldwide, against which exogenous phytohormone application might have beneficial effects (Table 2). ABA is known to play a major role in plant response to water deficit, with its level increasing under such stress. Travaglia et al. [6] provided a rare example of field research where the use of phytohormones was tested in open-field conditions, by investigating the effects of exogenous application of ABA on wheat plants under limited water availability. The results obtained over a 3-year trial showed that the exogenous application of ABA alleviated the impact of drought on plant growth and development. The ABA-treated plants consistently showed higher shoot biomass and photosynthetic activity, and their leaves stayed green for longer (up to a maximum of 10 days) as compared to untreated controls. Additionally, the contents of photosynthetic pigments chlorophyll A and carotenoids increased. However, the literature presents contrasting results on the role of ABA in prolonging photosynthetic activity; while, in some studies, ABA was found to inhibit the photosynthetic process [120,121], others, like Travaglia et al. [6] and Ivanon et al. [122], reported increased leaf carotenoid concentrations and a longer maintenance of the photosynthetic membrane integrity following exogenous application of ABA in barley.

Travaglia et al. [6] observed that ABA-treated wheat had 10% more closed stomata than controls at 7 h post phytohormone application, with similar values at 7 days, and even higher leaf conductance and transpiration rates at 21 days compared to untreated plants. These authors concluded that, when wheat is affected by a moderate water deficit, ABA treatment can exert long-term positive effects by maintaining better stomatal conductance than controls. However, all these effects of ABA had an impact on yield depending on the intensity of water stress; under a moderate deficit, ABA could increase yield by 20%, but under severe stress, no positive effects can be observed from phytohormone treatment.

Khan et al. [102] recently studied the impact of exogenously foliar-applied GA_3_, IAA and kinetin on yield parameters of rice under water stress imposed at panicle initiation. Results showed a significant increase in leaf chlorophyll content with +15% for GA and 5% increases for IAA compared to well-watered controls. The leaf proline and the soluble protein contents were also increased by GA, IAA and kinetin applications in water-stressed plants. According to Parida et al. [123], proline accumulation under water deficit was crucial for osmotic adjustment, protecting cell membranes and preventing chlorophyll losses. Proline accumulation under water deficiency was further augmented by GA_3_ and cytokinin applications, thus increasing the stress-adaptation strategy of plants, as observed by Travaglia et al. [6] in wheat.

Similar positive results were observed with GA_3_ treatments in maize under water stress [117], where water stress reduced overall plant biomass—affecting shoot growth more than root growth. Unlike in Khan et al. [102] in rice, GA_3_ was found to significantly alleviate these negative effects caused by water stress in maize by increasing plant biomass and water status. Although GA_3_ did not enhance proline accumulation in maize, it did increase the levels of nutrients such as Ca^2+^ and K^+^, the latter being a major osmolyte, thus its increased concentration due to GA_3_ treatment may be crucial in the plant adaptation to water deficit [124].

CK applications have been reported to increase drought tolerance by prolonging maintenance of the photosynthetic machinery and increased root growth relative to shoot growth [15,61,125]. Kumari et al. [64] found that foliar application of CKs in wheat under combined drought and high-temperature conditions lowered lipid peroxidation, improved relative water content by 27% and cell membrane stability by 11% and also enhanced chlorophyll content by 26%, with a 16% reduction in lipid peroxidation. Similarly, Gupta et al. [126] reported that the application of CK improved water status and chlorophyll content in water-stressed wheat as compared to untreated controls. Both studies by Kumari et al. [64] and Gupta et al. [126] reported an increased membrane stability and antioxidant enzyme activity, enhancing the membrane protection from ROS generated during stress conditions. Gupta et al. [126] also found that the increased levels of free proline in CK-treated wheat were associated with improved membrane integrity and water uptake.

Results of Dwivedi et al. [4] on screening of 25 wheat genotypes for water deficit stress tolerance and evaluating the effects of the exogenous CK application were consistent with the above-mentioned results. Indeed, CKs increased leaf water status by 11%, membrane stability by 5%, and total leaf chlorophyll content by 10% compared to water-stressed plants without CK application. Notably, the positive effect of CKs was more pronounced in genotypes that were more sensitive to water stress.

Few studies have explored whether regulating ethylene release in crops might alleviate drought damage (Table 2). Bergner and Teichmann [127] investigated the application of an ethylene-releasing compound (ethephon, i.e., 2-chloroethylphosphonic acid) and an ethylene biosynthesis inhibitor (AVG, i.e., amino-ethoxy-vinyl-glycine) on barley under soil water shortage. Ethephon application at jointing and anthesis promoted ethylene release, mirroring drought-induced effects such as reduced ear fertility, thus confirming increased ethylene production under environmental stress conditions. In contrast, the ethylene inhibitor AVG only partially mitigated drought-induced sterility, possibly due to its application on the main shoot apex.

Recent advancements have been made in managing the useful effect of the ACC deaminase enzyme provided by some plant-growth-promoting bacteria (PGPR) applied at the seed/soil/shoot level, which can reduce the internal ethylene levels, thereby improving stress tolerance in crops [21]. The use of PGPR can limit ethylene accumulation in plant tissues while also helping in exogenous phytohormone delivery. Indeed, it has been demonstrated that various bacterial strains can synthesize and transfer plant hormones that enhance plant tolerance to abiotic stresses [128,129].

The use of SA against drought has shown contrasting results depending on the application method. SA improved drought tolerance when applied by seed soaking before sowing—resulting in increased biomass, superoxide dismutase activity, and total chlorophyll content compared to untreated controls [130,131]. However, foliar application of SA has sometimes been associated with decreased drought tolerance [74], although some other studies on wheat and barley have reported positive outcomes, including increased antioxidant enzyme activity, chlorophyll content, and membrane stability [132,133].

**Table 2:** Phytohormones involved in plant response to abiotic stresses and experimental results on the effects of exogenous application under drought and salinity.

Phytohormone	Abiotic Stress Response Regulation of Plants	Effect of Exogenous Phytohormone Application on Crop Response to Abiotic Stress	
		DROUGHT	SALINITY
Cytokinin	Decreased CK levels during abiotic stress occurance. Transient elevation of CK levels with short-term stress [72]. Regulate shoot and root growth ratio. Modulate leaf enzymatic antioxidant activities [74]. Crosstalk with ABA: to regulate stress-response signaling and root growth [11]; reduction in CK content led to ABA hypersensitivity.	Extended maintenance of photosynthesis. Increased leaf relative water content (LRWC), chlorophyll content, membrane stability, root to shoot biomass ratio [15,64]. Decreased lipid peroxidation and induction of antioxidant enzyme activity [126].	Contrasting results have been reported [62].
Abscisic acid	Increased ABA levels during abiotic stress occurrence. Responsible for stomatal closure, regulation of transpiration and osmotic processes [31]. Maintenance of shoot growth in long-term responses to drought [83]. Induced leaf proline accumulation. Increased xylem water potential and ion accumulation cell vacuoles of roots during salinity events [71].	Prolonged canopy greenness. Increased chlorophyll and carotene content [6]. Maintenance of photosynthetic membrane integrity [122]. Decreased lipid peroxidation. Higher leaf conductance as long-term effect.	Reduced shoot Na^+^ and increased K^+^ concentration [112].
Ethylene	Increased ethylene release associated with stress symptoms. Crosstalk with ABA: shoot and root growth regulation; ethylene production regulated by ABA levels [97].	Application of ethylene inhibitors. Contrasting results: seldom completely reverses the effects of drought [86,127].	
Gibberellins	Increased levels during abiotic stress occurrence. Maintainance of plant growth [62,100].	Increased chlorophyll content and LRWC [102,127].	Positive effects on both cell division and elongation [117].
Salicylic acid	Increased SA levels during abiotic stress occurrence. Enhanced antioxidative capacity, accumulation of proline [103].		Increased cell division in apical meristem of seedling roots and decreased root Na^+^ [119,134].

### 4.2. Phytohormone Application and Salinity

Improving field crop tolerance to excessive soil salinity, primarily from NaCl, is crucial for ensuring productivity under saline stress and expanding crop cultivation in suboptimal environments. In rice, the role of ABA in promoting salinity tolerance was investigated by Gurmani et al. [112]. Previous studies have suggested a strong association between ABA application and the inhibition of Na^+^ accumulation in rice tissues [135]. Gurmani et al. [112] evaluated ABA as seed pre-treatment in rice under osmotic stress, both with and without silicate (Si)—a nutrient known to enhance resistance to abiotic stresses.

Exogenous ABA application significantly reduced shoot Na^+^ concentration, and the combination of ABA and Si resulted in a 57% greater reduction in Na^+^ accumulation compared to treatments with ABA or Si alone. As noted by Zhang et al. [136], salt tolerance in plants depends not only on controlling Na^+^ uptake but also on enhancing acquisition of K^+^, which is often suppressed by elevated external Na^+^ levels. In the study by Gurmani et al. [112], shoot K^+^ concentrations significantly increased under the ABA + Si treatment, which also promoted the net assimilation rate of plants.

Similarly, indole acetic acid (IAA) levels are known to rise in plant tissues under salt stress. Treating wheat seeds with IAA before sowing has been reported to mitigate the adverse effects of salinity on seed germination [137,138].

The role of cytokinins in salinity responses is often examined in light of their antagonistic relationship with ABA. Iqbal et al. [139] hypothesized that cytokinins could increase salt tolerance in wheat plants by interacting with other hormones, particularly auxins and ABA. Javid et al. [62] observed salinity-induced growth reduction preceded the decline in CK levels in sensitive varieties, suggesting that the effects of salinity may be mediated more by hormonal crosstalk than by CKs alone. While some studies report that exogenous CK application improves seedling growth under salinity [139], others have recorded exacerbated negative effects [62].

Additionally, experimental results are available on exogenous GA_3_ application in maize grown in soils with high salt concentrations (Table 2). Tuna et al. [116] evaluated the potential of GA_3_ sprayed on maize to alleviate the deleterious effects of salt stress. The inhibition of plant growth was found to be significantly reduced by GA_3_ treatment, primarily promoting proline and antioxidant accumulation in plant tissues. Similar findings were reported by Kaya et al. [117], who proposed that GA_3_’s positive effect on plant growth might be linked to its promotion of cell division and cell elongation. The leaf chlorophyll content was also increased by GA_3_ during salinity stress but did not reach the level of controls. An increased germination potential under saline conditions was also found by Radi et al. [140] in pre-soaked wheat seeds with GA_3_.

Salicylic acid (SA) plays a significant role in regulating the plant signaling under salinity stress (Table 2). Shakirova et al. [134] reported that SA increased the resistance of wheat seedlings to salinity by enhancing cell division in the apical meristem, thereby improving the plant growth. In maize under salt stress, Gunes et al. [119] tested the effect of SA incorporation into the soil at increasing doses of 14, 69 and 138 mg/Kg in potted plants and observed a decrease in NaCl accumulation in roots. SA application increased plant biomass in both saline and non-saline conditions, with a more pronounced effect under salinity stress. Lipid peroxidation was reduced, and the accumulation of Na^+^ and Cl^−^ significantly decreased with the application of SA compared to untreated controls. Fahad and Bano [118] are among the few authors who investigated the effect of SA applied by foliar spraying in saline fields; after SA application to maize plants at 40 days after sowing, they observed a mitigation of the adverse effects of salinity, such as improved root length, and moderate increases in fresh and dry root biomass and increased membrane stability and concentration of antioxidant mechanisms (superoxide dismutase, peroxidase, ascorbate peroxidase).

### 4.3. Phytohormone Application and Flooding

Research has primarily focused on exogenous cytokinin application to alleviate waterlogging damage in maize plants [109,110,141]. Studies have shown that cytokinin application can counteract the negative effects on plant growth and leaf chlorophyll content caused by waterlogging. Younis et al. [110] suggested that flooding-induced chlorosis may be partially due to cytokinin deficiency, as flooding reduces cytokinin transport from roots to shoots. The application of 6-BA in maize, wheat and barley under waterlogging has been found to increase dry biomass accumulation and enhance photosynthetic activity and grain-filling rates [109,142].

### 4.4. Phytohormone Applications and Heat Stress

In a recent study, Hays et al. [86] investigated the effects of 1-methylcyclopropene (1-MCP), an ethylene receptor inhibitor, on wheat plants subjected to heat stress. The heat treatment involved exposing wheat to 38 °C for 2 days at 10 days after pollination (DAP) with 1-MCP applied at 9 DAP. This pre-treatment effectively mitigated the reductions in kernel weight and kernel number per ear in a heat-sensitive wheat cultivar. These promising results suggest the possibility to exogenously regulate ethylene perception to limit stress-induced negative effects in various field crops. However, since the study by Hays et al. [86] was conducted on wheat in pots under environmentally controlled conditions, with the heat stress imposed only for 2 days and followed by a return to normal conditions, further research is needed to assess wheat responses to heat stress and evaluate the effects of 1-MCP application in field conditions under persisting stress conditions.

### 4.5. Phytohormonal Regulation of Leaf Senescence

Senescence represents the final developmental stage of plant cells, tissues and organs and involves the whole plant in the case of annual cereals. Leaf senescence is a genetically controlled process that remobilizes phloem-mobile nutrients from senescing parts to new growing sinks [18]. Various developmental and environmental factors contribute to plant senescence regulation, influencing the timing of both senescence and nutrient remobilization, from mature leaves (source organs) to the sink grains [15].

**Figure 2:**
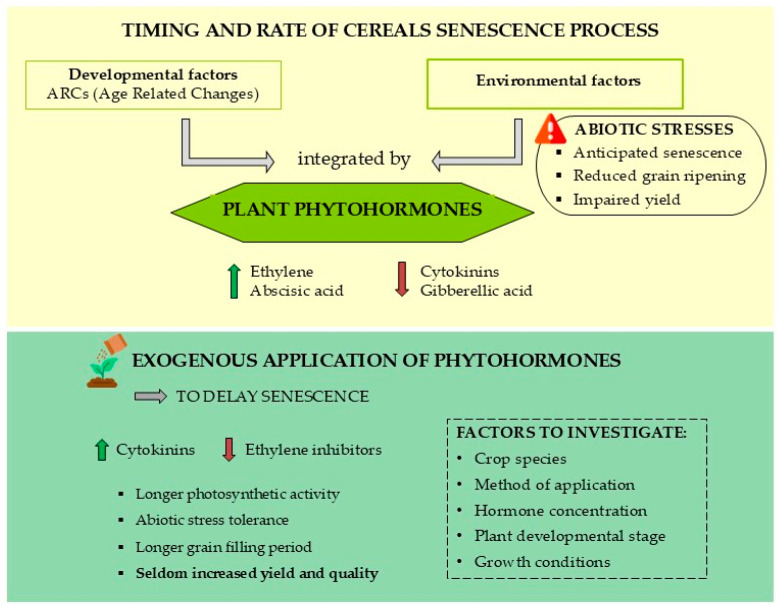
Role of phytohormones in regulating the senescence process in cereals and the potential effects of their exogenous application to delay plant senescence and enhance abiotic stress tolerance.

Plant phytohormones are widely known to regulate the leaf senescence process by integrating environmental signals and modulating both the onset and speed of senescence, thus affecting the nutrient content and biomass of the grains [9,143]. To date, only a few studies have investigated how phytohormones regulate the timing of age-related changes (ARCs), including the senescence process in crops. This section aims to provide a current overview of the insights gained so far (Figure 2).

Generally, leaf senescence in crops is associated with decreasing cytokinin levels and increasing ABA and ethylene levels [18,88,107,144]. Ethylene is considered a senescence-promoting phytohormone because its release increases during leaf and flower senescence. At the molecular level, ethylene has been shown to be involved in organized cell dismantling and the activation of nutrient recycling from senescing leaves to the other organs [88]. For instance, wheat ears progressively increased ethylene production from pre-anthesis at 0.76 nL g^−1^ FW h^−1^ to a peak of 1.53 nL g^−1^ FW h^−1^ at the hard dough stage, then it declined to a minimum of 0.10 nL g^−1^ FW h^−1^, as observed by Beltrano et al. [113].

Cytokinin content decreases during leaf senescence, suggesting it is a pivotal signal for initiating this physiological state [18]. During senescence, the transcript abundance of genes involved in CK biosynthesis, such as those coding for isopentyl phosphotransferases and cytokinin synthases, declines, while the mRNA abundance of genes involved in cytokinin degradation (e.g., cytokinin oxidases) increases [145]. Morris et al. [13] observed that the level of zeatin riboside (ZR) in wheat spikelets rose from anthesis to 2–3 days post anthesis (DPA), reaching a maximum of ~20 ng g^−1^ spikelet DM, before returning to basal levels by 7 DPA and then decreasing as senescence begins. Interestingly, the ZR peak occurring at 2–3 DPA coincides with the peak in endosperm cell division. According to Ha et al. [11], CKs regulate senescence through the movement of two CKs within the plant, i.e., trans-zeatin (tZ), which likely travels acropetally through the xylem, and isopentenyladenine (iP) that likely moves basipetally or systematically via the phloem.

In summary, the regulation of nutrient translocation, sink strength and grain yield is influenced by endogenous and environmental signals controlling CK movement within the plant [11]. Werner et al. [146] demonstrated that the enhanced root-specific expression of the CK-degrading gene *AtCKX* (*cytokinin oxidase/dehydrogenase*) led to lower foliar CK content and delayed leaf senescence. This effect was attributed to the increased root sink strength, indicating that cytokinins affect leaf senescence by altering the source–sink relations. This finding was also confirmed in rice plants by Yang et al. [147], who reported that grain and root CK contents play an essential role in regulating the grain-filling patterns. They observed an association between the changes in cytokinin (Z and ZR) content in the apical and basal rice spikelets and the grain-filling pattern. Genotypes exhibiting high grain-filling rates and percentages had elevated Z and ZR CK content in both apical and basal spikelets at early grain-filling stages. At this stage, CKs may induce the division of endosperm cells and thus create an increased sink force to enhance remobilization of assimilates. These findings suggest that boosting CK content in grains during the early filling stage could improve the grain-filling rate.

The phytohormone ABA integrates stress signals to regulate the induction of leaf senescence. As plants reach a certain developmental stage and face environmental stress, ABA mediates the signaling pathways that trigger senescence onset. Like ethylene, accelerated senescence in field crops is linked to increased ABA content [148].

Salicylic acid (SA) is involved in senescence regulation, mainly by balancing the negative and positive regulators of the process and affecting other phytohormone signaling pathways. SA has been reported to regulate the lipid metabolism during senescence by inducing autophagy within cytoplasmic components [149,150].

Gibberellic acid (GA_3_) is known to delay senescence, particularly as it declines in aging leaves. Some authors propose that it is not directly involved in senescence regulation, but it appears to have a role in antagonizing the effects of ABA [151].

Auxins have been identified as negative regulators of leaf senescence [152,153]. By directly modulating plant development, auxins influence senescence, while other phytohormones, such as ethylene, ABA and SA, primarily respond to environmental signals and stress [18].

There is growing evidence that different phytohormonal pathways interact antagonistically or synergistically to regulate leaf senescence. For instance, ethylene and auxins were found to act antagonistically; as the level of auxins in leaves decreases during senescence, the sensitivity of tissues to ethylene increases, promoting higher ethylene biosynthesis. Meanwhile, ethylene inhibits the auxin synthesis and transport, while enhancing auxin degradation [88].

It is widely believed that an effective strategy for boosting field crop production is to promote the delay of leaf senescence by extending the photosynthetic activity of the flag leaf in wheat to maintain the supply of assimilates to the grains for a longer period and potentially increase yield [154,155,156]. To achieve this, stay-green mutants have been developed across multiple species, offering various degrees of delayed senescence. The benefits of these phenotypes with extended greenness, especially under drought conditions, have been investigated in sorghum and maize [9]. This has led to growing interest in delayed leaf senescence for other cereal crops like wheat and barley, where it may be important for abiotic stress resistance [157,158]. Wheat mutants with a stay-green phenotype exhibit extended photosynthesis, improved stability of thylakoid membranes, and enhanced antioxidant capacity [154,159,160].

Nevertheless, the potential of the stay-green mutants to increase yield remains debated [161,162,163]. Some studies show that certain stay-green cultivars or species increase grain yield by delaying senescence [154,164], whereas others indicate that delayed senescence may be linked to a limitation in grain sink capacity, resulting in decreased grain weight [165,166]. There is no clear evidence of the potential of stay-green mutants to ensure higher grain yield against environmental stress occurring during grain filling. Hence, exogenous application of phytohormones may provide new avenues to attain similar results to stay-green cultivars while ensuring higher tolerance to abiotic stress and improving yield.

## 5. Phytohormone Applications for Senescence Regulation

The application of exogenous phytohormones to delay senescence in herbaceous crops has received increasing research interest in recent decades, although scientific evidence remains limited. In this section, we review the main experimental findings available in the literature, as summarized in Table 3.

Cytokinins have been identified as key regulators of senescence [40,146], and their exogenous application has been found to retard senescence. Xie et al. [167], for instance, studied the effects of exogenous ZR and ABA on photosynthetic characteristics of wheat ears cultured in vitro. Over 11 days of culture with continuous exogenous phytohormone treatment, the chlorophyll content in the flag leaf significantly declined under ABA treatment but increased significantly under ZR. The photosynthetic rate at 11 days after culture (DAC) was also significantly increased by ZR treatment, although no significant differences were found in grain weight or grain protein content at harvest.

Conversely, Yang et al. [84] observed increased stay-green characteristics, higher grain-filling rate, and more endosperm cell division following the application of 6-benzyladenine (6-BA) on winter wheat, concurrently with +2–6% of grain yield depending on the variety. Similarly, with the application of exogenous cytokinins, other authors reported an average +12% increase in grain yield in wheat and barley [168,169,170], and +4% to +8% in rice, depending on the investigated variety [171]. Morris et al. [13] found that spraying maize with benzyladenine (BA) at flowering increased total yield and kernel number compared to untreated controls, with an increase to a maximum of +30%. More recently, Ren et al. [109] applied 6-BA on maize plants post waterlogging and reported delayed leaf senescence, increased chlorophyll content and significantly increased grain yield (up to +17%). Similar results were found by Gao et al. [111], who highlighted the role of cytokinins in improving the source–sink balance in maize by spraying 6-BA at the tasselling stage, which hampered leaf senescence and increased photosynthesis and the number of kernel endosperm cells, leading to a higher grain-filling rate and higher kernel weight. Since the endosperm accounts for approximately 80% of the kernel weight [171], higher levels of CKs found in the endosperm of developing kernels may explain how exogenously applied 6-BA can enhance cell division and improve the capacity of the kernel sink, thus improving grain weight. In accordance, cytokinin concentrations in wheat spikes at anthesis were positively correlated with grains per m^2^, suggesting that CK application at this stage offers scope to raise floret fertility [172] as well as grain number and yield potential [173].

However, the positive effects of CK application, as pointed out by Koprna et al. [174], are heavily influenced by factors such as the species, method of CK application, developmental stage and growth conditions. Yang et al. [107,147,175] investigated exogenous application of ABA on wheat and found it accelerated the grain-filling rate, thereby boosting carbon remobilization and improving the grain weight. In contrast, Xie et al. [167] found that, while ABA similarly affected nutrient remobilization in wheat, it reduced grain weight. Yang et al. [107] proposed that ABA hastens senescence by raising ethylene synthesis or sensitivity, which triggers plant senescence.

Ethylene has also been examined for its role in cereal senescence dynamics and grain weight. Beltrano et al. [113,114] exogenously applied ethylene (ethephon) and ethylene inhibitors (AVG and silver thiosulfate) to wheat ears post anthesis and observed that ethylene accelerated senescence and lowered final grain dry weight, whereas ethylene inhibitors delayed chlorophyll degradation, extended floret/grain metabolism and enabled more assimilate accumulation in grains, thus resulting in increased grain dry weight. Other compounds can similarly delay senescence by ethylene regulation. Grossmann et al. [176] studied the secondary effects of the fungicide kresoxim-methyl (with a mitochondrial respiration inhibiting effect) on phytohormonal changes in wheat, and the application of this product was found to be associated with delayed leaf senescence and improved water-conserving effect. The inhibition of ethylene biosynthesis through the reduction of endogenous 1-aminocyclopropane-1-carboxylic acid (ACC, ethylene precursor) synthase activity was also observed. Contrastingly, the levels of cytokinins increased by 3-fold with an ethylene reduction of up to 60%. The treatment with kresoxim-methyl, then, affected ABA signaling and the ethylene–CK balance by favoring ZR-type cytokinins and delaying leaf senescence.

Plant response to exogenous hormone application is also dependent on nitrogen (N) availability. N fertilization management is crucial for maintaining canopy longevity during grain filling and to enhance grain yield and quality [142,155]. The interaction between N supply, phytohormone application and N remobilization remains a key yet understudied area. A strong correlation between CK levels and N has been highlighted, including demand for and acquisition, and remobilization of N. While nitrogen could regulate CK biosynthesis and degradation, CKs in turn mediate the absorption, assimilation and metabolism of nitrogen. Sýkorová et al. [177] suggested that wheat grown under a low N supply may be more responsive to increased cytokinin levels achieved by exogenous application. Luo et al. [108] examined the effects of the foliar application of 6-BA on wheat under three varying N rates: low (0 kg ha^−1^), normal (240 kg ha^−1^) and high (360 kg ha^−1^) and found that, while the highest total nitrogen in the flag leaf occurred under the 6-BA × high N treatment, the highest grain weight occurred under 6-BA × normal N treatment.

According to current literature, delaying senescence can yield mixed outcomes regarding the balance between grain biomass and quality, partly due to the well-known inverse correlation between yield and grain protein content (GPC) in cereals. Most research on phytohormones’ effects on N metabolism and grain protein accumulation during grain filling was investigated mainly with ABA and cytokinins. Xie et al. [167], using a wheat ear culture system, studied the effects of exogenous ABA and CKs on glutamate pyruvate transaminase (GPT) and glutamine synthetase (GS), two key enzymes in amino acid conversion and protein accumulation [178]. ABA treatment increased the GPT and GS activity, accelerating nitrogen remobilization to grains, whereas cytokinins reduced GS and GPT activity, delaying amino acid conversion into grain protein. A higher protein content was found in wheat ears subjected to ABA treatment; cytokinins, despite raising the photosynthetic rate in the flag leaf (increased levels till 11 DAC), did not improve grain yield and quality.

Criado et al. [15] examined the N remobilization process in wheat by evaluating the impact of exogenous 6-BA on protein synthesis and chloroplast structure. They found that exogenous 6-BA treatment increased protein, chlorophyll, Rubisco, sugar and starch concentrations in older fully expanded leaves, while it decreased protein and chlorophyll concentrations in younger expanding leaves. The authors, aligning with Roitsch and Ehneß et al. [179], suggested that the cytokinins’ mode of action involves inhibition of amino acid and sugar export to phloem. Moreover, in the study performed by Criado et al. [15], BAP treatment has been shown to inhibit protein degradation and increase chloroplast size, mainly through stromal swelling, leading to larger starch grains. In a study carried out on transgenic tobacco leaves, it was pointed out that increased CKs activate invertases, thereby reducing the amount of sucrose (a mobile sugar), while increasing the availability of glucose and fructose, thus inducing the leaf to stay green longer (due to the availability of prompt energy) [180]. Collectively, these findings suggest that cytokinins maintain the sink activity of the older leaves by inhibiting amino acid, glucose and sucrose export to the phloem, thus redirecting the incoming nitrate into these leaves, and increasing protein concentration. Several studies highlighted the association between an early and/or efficient nutrient remobilization and a higher grain protein concentration [181]. Additionally, this process has been associated with an increased concentration of beneficial micronutrients such as Fe and Zn [9,182].

Ultimately, the impact of phytohormone application on the nutrient remobilization to grains during grain filling remains an open question. There are contrasting results in the literature regarding the effects of CK treatment on yield and grain protein content of field crops. This strongly suggests the need for further research on how phytohormone application, nitrogen availability and nutrient remobilization interact with factors such as timing of phytohormone application, concentration in the applied solution, crop species and prevailing environmental situations for gaining better yield and quality.

**Table 3:** The role of three main phytohormones involved in the senescence process of annual crops, i.e., cytokinins, abscisic acid and ethylene.

Phytohormone	Senescence Process Regulation	Agronomic Effects of Exogenous Phytohormone Application	
Cytokinin	Decreased CK contents in senescing leaves. Regulates senescence onset and process by mediating the movement of tZ acropetally through the xylem and of iP basipetally through the phloem [11]. Regulates senescence by increasing the root sink strength [146].	**Delay of leaf senescence** Prolonged photosynthetic activity, increased endosperm cell division rate and delayed nutrient remobilization to grains. Inhibition of amino acid and sugar export to the phloem. It maintains the sink activity of older leaves: protein, chlorophyll and Rubisco increased in older leaves and decreased in young leaves [15].	
		**Yield and/or gpc improved**: Wheat and barley: +2–15% yield [84,168,169,170]. Rice: +4–8% yield [170]. Maize: +3–30% yield [13,109,111].	**Yield and gpc not improved**: Wheat: –16% yield [167].
Abscisic acid	Increased ABA content associated with accelerated senescence; it integrates stress signal to induce senescence onset. Negatively regulates tolerance responses through inhibition of stomatal closure to induce senescence [69].	**Acceleration of leaf senescence**: It accelerates grain-filling rate and N remobilization to grains, increases activity of GS and GPT enzymes for conversion of amino acids to storage proteins [167]. Crosstalk with ethylene: senescence acceleration by increasing ethylene synthesis or sensitivity [107].	
		**Yield and/or gpc improved**: Wheat: +3% yield [107].	**Yield and gpc not improved**: Wheat: –31% yield [167].
Ethylene	Senescence-promoting hormone; increased release during leaf senescence. Cell-dismantling regulation. Activation of nutrient remobilization.	Application of ethylene inhibitor compounds to **delay leaf senescence** Delayed chlorophyll degradation and grain filling [113,114].	

## 6. Conclusions

Phytohormones regulate the perception of stress cues and the relay of signals that activate adaptive strategies. They modulate senescence—positively or negatively—by integrating developmental and environmental signals, thus ensuring senescence timing and progression are adapted to environmental conditions. Exogenous application of phytohormones in field crops has the potential to strengthen these stress-adaptation responses and alter plant growth and developmental pathways. Although the number of studies assessing the effects of phytohormone application on field crops has increased over recent decades, the topic remains poorly investigated, with most of the experiments being performed under environmentally controlled conditions.

Further research is needed to investigate phytohormone treatments under open-field conditions and in various crops by comparing contrasting application timing and different phytohormone concentrations. The collective findings reviewed here indicate that phytohormones hold significant potential for improving abiotic stress tolerance and yield in many field crops. Additionally, advancing our understanding of the molecular mechanisms involved in phytohormone regulation is essential to increase our understanding of the potential of this agricultural practice to enhance field crop productivity. However, currently there is no conclusive evidence on the optimal time and concentrations of hormone applications, and there is a lack of species-specific information. Thus, there is a consistent need to expand our knowledge of the effects of different treatments on field crop growth and development and to determine how these effects can translate into agronomic benefits.

## Figures and Tables

**Table 1:** Materials and methods of investigated exogenous phytohormone application in field crop trials. Treatment types: 1 = pre-sowing seed treatment; 2 = foliar distribution during vegetative growth; 3 = post-anthesis application to delay senescence.

Phytohormone	Treatment Type	Crop	Phytohormone Concentration (mg/L)	Application Time/Method	Target Stress/Aim	Reference
Cytokinin	1	Wheat	10 6-BAP	Seed pre-treatment	Drought and heat	[64]
	3	Wheat	8.61 kinetin	Sprayed from 9 DPA for 5 consecutive days	Drought	[107]
	3	Wheat	25 6-BA	Sprayed daily for 3 days after anthesis	Yield increase	[108]
	3	Wheat	10 6-BA	Sprayed daily for 3 days after anthesis	Heat	[84]
	2	Maize	100 6-BA	Sprayed the day after waterlogging stress	Waterlogging	[109]
	2	Maize	50 kinetin	Sprayed on waterlogged plants	Waterlogging	[110]
	3	Maize	25 6-BA	Sprayed at tasselling for 3 consecutive days	Yield increase	[111]
Abscisic acid	1	Rice	2.46 ABA	Seed pre-treatment	Salinity	[112]
	2–3	Wheat	300 ABA	2 treatments: at shoot enlargement and anthesis	Drought	[6]
Ethylene	3	Wheat	8.06 AVG	Weekly from anthesis till hard dough stage	Yield increase	[113,114]
	2	Wheat	1 mg/Kg 1-MCP	Sprayed once before heat stress occurrence	Heat	[86]
Gibberellins	1	Wheat	Not cited	Seed pre-treatment	Yield increase	[115]
	2	Maize	25–50–100 GA_3_	Weekly from 10 till 45 DAG	Salinity	[116,117]
	2	Rice	3.46 GA_3_	At panicle initiation	Drought	[102]
Salicylic acid	2	Maize	1.38 SA	Leaf spraying at 40 DAS	Salinity	[118]
	1	Barley	138 SA	Seed pre-treatment	Salinity	[104]
	-	Maize	14–69–138 SA	Incorporated into the soil (pot trial)	Salinity	[119]

## Data Availability

No new data were created or analyzed in this study.
